# On the impact of modelling assumptions in multi-scale, subject-specific models of aortic haemodynamics

**DOI:** 10.1098/rsif.2016.0073

**Published:** 2016-06

**Authors:** Jordi Alastruey, Nan Xiao, Henry Fok, Tobias Schaeffter, C. Alberto Figueroa

**Affiliations:** 1 Department of Biomedical Engineering, King's College London, St Thomas’ Hospital, London, UK; 2 Department of Clinical Pharmacology, King's College London, St Thomas’ Hospital, London, UK; 3 Department of Bioengineering and Surgery, University of Michigan, Ann Arbor, MI, USA

**Keywords:** haemodynamics, pulse wave propagation, subject-specific modelling, magnetic resonance imaging, applanation tonometry

## Abstract

Simulation of haemodynamics has become increasingly popular within the research community. Irrespective of the modelling approach (zero-dimensional (0D), one-dimensional (1D) or three-dimensional (3D)), *in vivo* measurements are required to personalize the arterial geometry, material properties and boundary conditions of the computational model. Limitations in *in vivo* data acquisition often result in insufficient information to determine all model parameters and, hence, arbitrary modelling assumptions. Our goal was to minimize and understand the impact of modelling assumptions on the simulated blood pressure, flow and luminal area waveforms by studying a small region of the systemic vasculature—the upper aorta—and acquiring a rich array of non-invasive magnetic resonance imaging and tonometry data from a young healthy volunteer. We first investigated the effect of different modelling assumptions for boundary conditions and material parameters in a 1D/0D simulation framework. Strategies were implemented to mitigate the impact of inconsistencies in the *in vivo* data. Average relative errors smaller than 7% were achieved between simulated and *in vivo* waveforms. Similar results were obtained in a 3D/0D simulation framework using the same inflow and outflow boundary conditions and consistent geometrical and mechanical properties. We demonstrated that accurate subject-specific 1D/0D and 3D/0D models of aortic haemodynamics can be obtained using non-invasive clinical data while minimizing the number of arbitrary modelling decisions.

## 1. Introduction

Computational modelling of cardiovascular dynamics has received notable attention over the last two decades. The modelling approaches range from lumped parameter, zero-dimensional (0D) models of the circulation [1,2], to one-dimensional (1D) models of blood pressure and flow propagation [3–7], to three-dimensional (3D) fluid–structure interaction techniques [8–11]. Each approach has its own merits and limitations. Lumped parameter methods provide a computationally inexpensive, mathematically accessible and intuitive framework to study whole-system dynamics. However, they are not suitable for studying pulse propagation phenomena or complex flows. Nonlinear 1D methods can accurately describe pulse wave propagation phenomena in extensive vascular networks while keeping the computational cost down. However, these methods are not appropriate to describe complex 3D flow features, like those observed in stenosis and aneurysms. Lastly, 3D methods are capable of representing complex flows, wave propagation and blood flow–vessel wall interactions. On the downside, a 3D approach is computationally expensive.

Irrespective of the modelling approach, clinical measurements are required to personalize the geometry, material properties (e.g. vessel wall stiffness) and inflow and outflow boundary conditions of the computational framework. *In vitro* studies have shown the ability of 1D and 3D modelling to reproduce the main features of pressure and flow waveforms in the aorta and larger systemic arteries, if accurate measurements of *in vitro* data are available (e.g. [12,13]). There are, however, numerous challenges in acquiring haemodynamic data *in vivo*, particularly when using non-invasive techniques. For instance, magnetic resonance imaging (MRI) is limited by the acquisition time of certain sequences, their spatial and temporal resolution, and the variability in the physiology of the subject over the imaging study [14]. Ultrasonic techniques are affected by tissue signal attenuation and thus cannot reliably be used to measure flow in deep vessels [15]. Lastly, sphygmomanometry or tonometry are operator dependent, usually restricted to superficial vessels, and arguably introduce perturbations in the measured waveform, as they involve applying pressure in the region of interest [16]. Additional challenges arise from using measurements derived from different sources, which is common clinical practice. Indeed, these measurements are often not concurrent in time and, hence, subject to potential variability in heart rate (HR) and haemodynamic state, and may violate physical principles such as mass conservation.

The above limitations in data acquisition often result in insufficient information to determine the material and boundary condition parameters required for the simulation. Therefore, numerous modelling assumptions are usually made that are not directly informed by data. These modelling assumptions rely on morphometric considerations, such as more flow goes to larger vessels, specification of non-reflective outflow boundaries, and population-based rules for assigning tissue stiffness or pulse wave velocity.

In this article, we present a computational study of aortic pulse wave haemodynamics in a young healthy volunteer using multi-scale 1D/0D and 3D/0D frameworks and a rich array of non-invasive haemodynamic data, including magnetic resonance angiography, phase-contrast MRI, dynamic area and tonometry measurements. The clinical data and the set-up of the 1D and 3D frameworks were chosen to minimize the number of arbitrary modelling assumptions on boundary conditions and material parameters. This article has three main objectives: (i) to investigate the impact of different modelling assumptions on the simulated waveforms, (ii) to verify haemodynamic predictions obtained with 1D and 3D computational frameworks against clinical data, and (iii) to identify inconsistencies in the clinical data and discuss strategies to mitigate their impact on the simulation workflow.

## 2. Material and methods

### 2.1. *In vivo* data: measurement and post-processing

We acquired a series of non-invasive haemodynamic measurements on a young healthy male volunteer (age 27 years, weight 80 kg, height 188 cm) using a 1.5 T MRI scanner (Philips Achieva, Philips Healthcare) and applanation tonometry (SphygmoCor system, AtCor, Australia) at Guy's Hospital, London, UK. All measurements were taken at rest with the volunteer in the supine position. We followed a protocol approved by the local ethics committee and the volunteer provided written informed consent. A brief description of the data acquisition technology is given in appendix A.

#### 2.1.1. Arterial geometry

**Figure 1.**
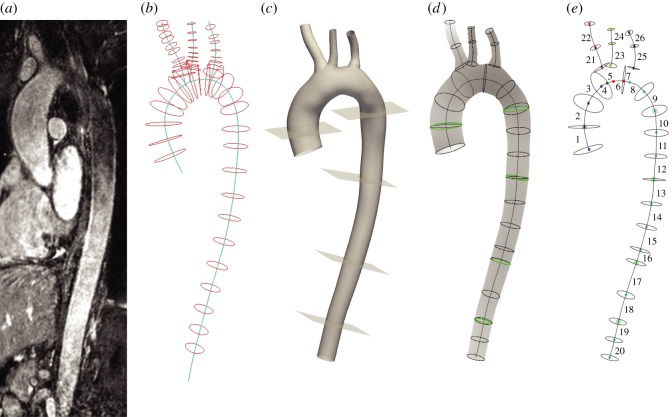
(*a*) Maximum intensity projection of the thoracic aorta from the anatomy scan. (*b*) Centrelines and contours used for reconstruction of the 3D geometry. (*c*) 3D geometry and planes corresponding to the MRI flow/area measurements. (*d*) Centrelines and contours (black) used for the definition of the 1D geometry and the contours (green) corresponding to the MRI measurements. (*e*) Segments of the 1D geometry used for assigning length, radius and pulse wave velocity as given in table 1.

A non-contrast, free-breathing, two-phase (systole and diastole) 3D SSFP scan (TE: 3 ms, TR: 5 ms, flip angle: 90°, field of view: 320 mm, slice thickness: 2 mm) was carried out on the MRI scanner. The diastolic phase was used to reconstruct the 3D aortic geometry and generate finite-element meshes for the 1D and 3D simulations (figure 1), as detailed in §2.2.1.

**Table 1.** Geometric properties and pulse wave velocities of the upper aorta model measured non-invasively in a young healthy volunteer. 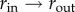: diastolic cross-sectional radii at the inlet and outlet of the arterial segment. 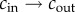: pulse wave speed (at diastolic pressure) at the inlet and outlet of the arterial segment calculated using either the foot-to-foot or *QA*–loop method.

arterial segment	length (cm)	*r*_in_ → *r*_out_ (mm)	*c*_in_ → *c*_out_ (m s^−1^)	
			foot to foot	*QA*–loop
1. aorta I	1.94	12.4 → 13.2	4.56 → 4.56	4.56 → 4.42
2. aorta II	2.06	13.2 → 13.2	4.56 → 4.56	4.42 → 4.42
3. aorta III	2.00	13.2 → 12.8	4.56 → 4.56	4.42 → 4.49
4. aorta IV	0.58	12.8 → 12.3	4.56 → 4.56	4.49 → 4.56
5. aorta V	0.57	12.3 → 11.8	4.56 → 4.56	4.56 → 4.66
6. aorta VI	0.85	11.8 → 11.1	4.56 → 4.56	4.66 → 4.81
7. aorta VII	0.48	11.1 → 10.9	4.56 → 4.56	4.81 → 4.86
8. aorta VIII	1.52	10.9 → 10.7	4.56 → 4.56	4.86 → 4.91
9. aorta IX	2.06	10.7 → 10.6	4.56 → 4.56	4.91 → 4.93
10. aorta X	1.94	10.6 → 10.0	4.56 → 4.56	4.93 → 5.08
11. aorta XI	2.00	10.0 → 9.3	4.56 → 4.56	5.08 → 5.27
12. aorta XII	1.97	9.3 → 9.1	4.56 → 4.56	5.27 → 5.31
13. aorta XIII	2.03	9.1 → 8.8	4.56 → 4.56	5.31 → 5.41
14. aorta XIV	2.00	8.8 → 8.6	4.56 → 4.56	5.41 → 5.46
15. aorta XV	2.00	8.6 → 8.5	4.56 → 4.56	5.46 → 5.50
16. aorta XVI	1.10	8.5 → 8.3	4.56 → 4.56	5.50 → 5.57
17. aorta XVII	2.90	8.3 → 7.8	4.56 → 4.56	5.57 → 5.76
18. aorta XVIII	2.41	7.8 → 7.5	4.56 → 4.56	5.76 → 5.87
19. aorta XIX	1.59	7.5 → 7.5	4.56 → 4.56	5.87 → 5.85
20. aorta XX	1.56	7.5 → 7.5	4.56 → 4.56	5.85 → 5.84
21. brachiocephalic I	2.00	7.0 → 4.5	4.56 → 4.56	6.06 → 7.54
22. brachiocephalic II	2.20	4.5 → 4.3	4.56 → 4.56	7.54 → 7.77
23. left carotid I	2.00	5.1 → 3.1	4.56 → 4.56	7.10 → 9.18
24. left carotid II	1.23	3.1 → 2.9	4.56 → 4.56	9.18 → 9.43
25. left subclavian I	2.00	5.3 → 3.5	4.56 → 4.56	6.97 → 8.64
26. left subclavian II	1.31	3.5 → 3.4	4.56 → 4.56	8.64 → 8.67

#### 2.1.2. Volume flow rate and luminal area pulse waves

**Figure 2.**
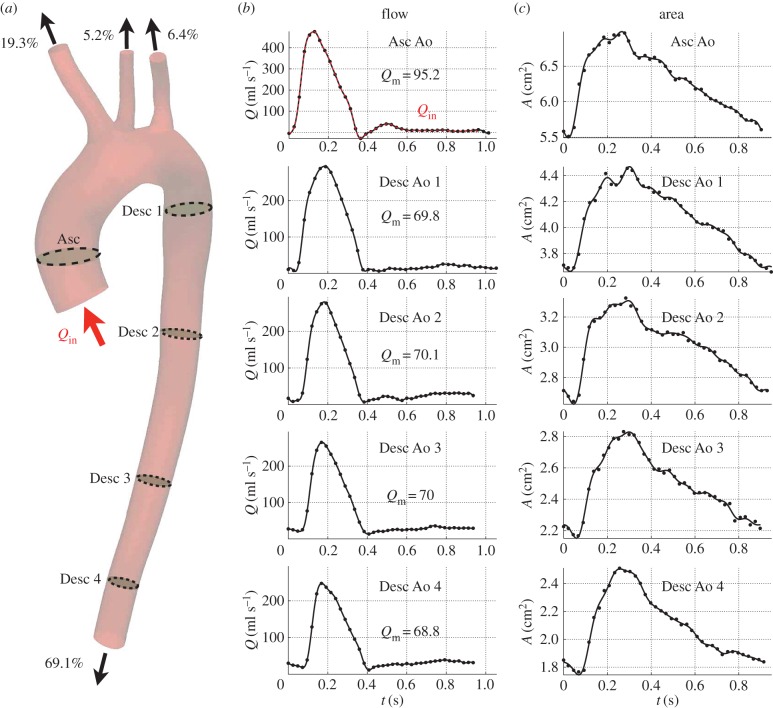
(*a*) Geometry of the human upper aorta acquired by MRI. (*b*) Blood flow waveforms measured by PC-MRI at the five planes highlighted in the aortic geometry (*a*). (*c*) Luminal area waveforms obtained from 2D cine SSFP MRI on the same planes. The 40 phases of the flow and area signals are shown in dots and the post-processed waves in solid lines. Mean flow rates (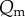) were calculated in ml s^−1^ from the post-processed flow waveforms. The flow waveform measured at the ascending aorta (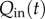) was prescribed as inflow boundary condition at the aortic root. The percentage of mean flow 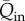 leaving each terminal vessel is indicated next to the outflow arrows (*a*).

Volume flow rate waveforms (figure 2*b*) were obtained at five aortic locations (figure 2*a*) using gated PC-MRI (TE: 3 ms, TR: 5 ms, flip angle: 15°, field of view: 350 mm, slice thickness: 8 mm, Venc: 2 m s^−1^). We used flash angiography images to ensure that measurement planes were approximately perpendicular to the aortic axis. Dynamic area images were acquired using gated two-dimensional (2D) cine SSFP MRI on the same planes (TE: 1 ms, TR: 4 ms, flip angle: 50°, field of view: 370 mm, slice thickness: 8 mm) to obtain aortic luminal area waveforms (figure 2*c*). Flow measurements to the upper branches were obtained via PC-MRI in a separate study in the same volunteer. This prior information was used to determine the flow distributions in the supra-aortic vessels (figure 2*a*). All flow and area measurements were acquired with 40 phases. The volunteer held his breath during each measurement to minimize movement artefacts.

The automatic image segmentation and analysis tools of ViewForum (Philips Healthcare) were used to segment luminal cross-sectional areas from the dynamic area images. The software GTFlow (GyroTools LLC) was used to analyse the PC-MRI data and reconstruct flow waveforms.

#### 2.1.3. Pressure pulse waves

**Figure 3.**
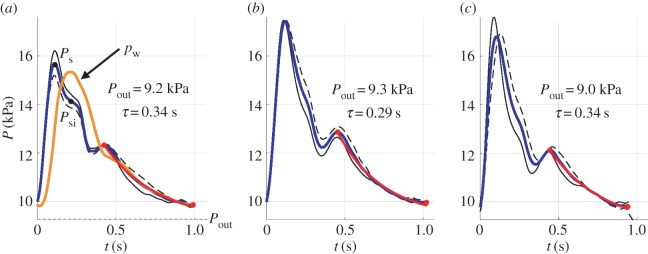
Ensemble averaged pressure waveforms (blue lines) in the (*a*) left common carotid, (*b*) left brachial and (*c*) right radial arteries obtained from *in vivo* tonometry measurements before (solid black lines) and after (dashed lines) placing the volunteer in the MRI scanner. (*a*) The space-independent pressure, 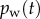, calculated by equation (B 3) with the parameters of the ‘best pressure’ model (orange line). The exponential fit of the form given by equation (2.7) to the decay in pressure in the last part of diastole is shown in red lines. The asymptotic pressure 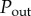 and time constant 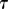 of the fit are provided in each panel. For the carotid wave, the pressures required to calculate 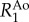 using equation (2.15) are displayed: the systolic pressure (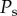) and the pressure at the systolic inflection point (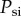) using black dots, and 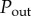 using a dashed horizontal line.

Blood pressure waveforms were recorded in the left common carotid, left brachial and right radial arteries using applanation tonometry performed by an experienced operator using a SphygmoCor system (figure 3). Measurements were taken in a quiet environment just before and after the MRI study. A sliding stretcher was used to move the volunteer to maintain the HR as constant as possible.

For each measurement site, at least 10 cardiac cycles were obtained at a sampling frequency of 128 Hz and ensemble averaged by the SphygmoCor system. Waveforms that did not meet the in-built quality control criteria in the SphygmoCor system were rejected. All pressure waveforms were calibrated with brachial blood pressure measured in triplicate by a validated oscillometric method (Omron 705CP, Omron Health Care, Japan). Figure 3 shows the resulting left carotid, left brachial and right radial pressure waveforms calculated by averaging the corresponding measurements before and after placing the volunteer in the MRI scanner.

### 2.2. Numerical formulations

Detailed descriptions of the 1D/0D and 3D/0D formulations used in this study are given in [7] and [11], respectively. Here we summarize their main assumptions and describe their input physical parameters and simulated haemodynamic quantities that are relevant to this study.

In the 1D/0D framework, the aortic geometry was represented by interconnected segments, each one modelled as a deformable vessel with properties depending on a single axial coordinate *x*. The following haemodynamic quantities were simulated as a function of *x* and time *t*: the cross-sectional luminal area, 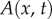, and the cross-sectional averages of the axial blood flow velocity, 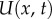, and pressure, 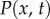. The axial velocity was assumed to have an axisymmetric profile.

In the 3D/0D formulation, vessel wall displacement and blood velocity were simulated as 3D vectors varying with time and space. Blood pressure was modelled as a function of time and the three spacial coordinates. Vessel wall displacements were described as a function of the blood velocities and pressures at the fluid–wall interface, using an ‘enhanced’ membrane formulation with a fixed fluid domain and linearized kinematics of the vessel wall.

In both the 1D/0D and 3D/0D models, blood was assumed to be incompressible and Newtonian, with a constant density 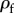 and viscosity *μ*. The arterial wall was modelled as a thin, incompressible, homogeneous, isotropic, linear elastic, impermeable membrane characterized by an elastic modulus *E*, Poisson's ratio 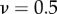 and thickness *h*. In the 1D model, the membrane was also assumed to deform axisymmetrically, each cross section independently of the others. The following explicit algebraic relationship between *P* and *A* (or *tube law*) was used [4]:
2.1
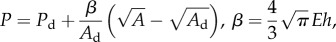
where 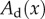 is the luminal area at diastolic pressure (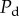) and 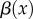 accounts for the elastic material properties of the arterial wall. This tube law leads to the following expression for the pulse wave velocity (*c*) [7]:
2.2
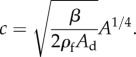
For 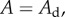 we have
2.3
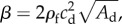
which relates the elastic parameter *β*(*x*) required to run the 1D model with the pulse wave velocity at diastolic pressure, 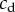, calculated from the *in vivo* data.

In the 3D formulation, no assumptions regarding axisymmetry were made. The arterial wall was modelled as a thin pre-stressed membrane with the following parameters, in addition to the elastic modulus and Poisson's ratio: density 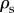 and transverse shear factor *k* [11]. The mechanical behaviour of the external tissue was modelled using stiffness (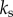) and damping (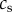) coefficients. This was important to account for the tethering exerted by the external tissue on the arterial walls and eliminate spurious and non-physiological oscillations [17].

#### 2.2.1. Three-dimensional and one-dimensional mesh generation

The 3D aortic geometry was reconstructed from the magnetic resonance angiography data (figure 1*a*). Centrelines were first defined for the aorta and three supra-aortic vessels (figure 1*b*). Then, perpendicular to the centrelines, a series of 2D contours were created by segmenting the lumen of the vessel (figure 1*b*). These contours were used to generate a 3D parametric surface (figure 1*c*). A finite-element mesh (159 392 linear tetrahedral elements) for the 3D simulations was created using the MeshSim library (Simmetrix Inc., NY, USA).

To obtain the 1D network topology, we generated four centrelines (figure 1*d*): one through the aorta and three through the supra-aortic vessels. Subsequently, cross sections were computed perpendicularly to the centrelines and spaced 20 mm apart, defining the 26 segments that form the 1D model mesh (figure 1*e*). Table 1 shows the length and radii at the inlet and outlet of each 1D model segment. Finite-element meshes for each segment were created with quadrature rules and polynomial functions of order 3.

#### 2.2.2. Boundary conditions

Inlet and outlet boundary conditions were chosen to be consistent between the 1D and 3D schemes. At the inlet, we prescribed the flow waveform, 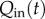, measured by phase-contrast MRI, with the time period corrected as described in §2.4 (figure 2*b*, colour line). This waveform was repeated for at least four cardiac cycles in the 1D simulations and 10 cardiac cycles in the 3D simulations to achieve periodic solutions. The velocity profile at the inlet of the 3D domain was assumed to be axisymmetric in agreement with the 1D formulation profile.

**Figure 4.**
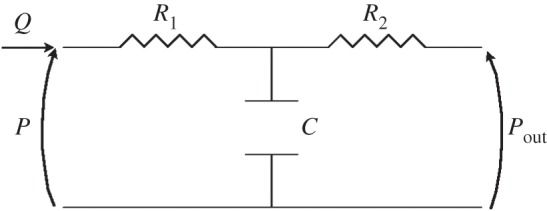
One-dimensional analogous electrical circuit of the three-element Windkessel model relating the outgoing flow *Q* to the pressure *P* at the end point of each terminal branch in the 1D and 3D models.

The outlets of the descending aorta and three supra-aortic arteries were coupled to three-element Windkessel models. These 0D electrical circuit analogues of the downstream vasculature consist of proximal resistance, 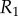, connected in series with a parallel combination of a distal resistance, 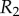, and a compliance, *C* (figure 4). A non-zero outflow pressure, 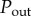, was considered, which can be interpreted as the pressure at which flow to the microcirculation ceases.

### 2.3. Calculation of model parameters from non-invasive *in vivo* data

In addition to the aortic geometry and the inlet flow boundary condition described above, material and outflow boundary parameters are required to run 1D and 3D simulations. This section describes the strategy for specifying the latter considering the available non-invasive data and a number of different modelling approaches. In particular, the following three independent parameters must be characterized: (i) pulse wave velocity at diastolic pressure (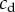), (ii) outflow pressure (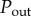), and (iii) proximal resistance (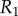) for each Windkessel. We explored two different strategies to determine the value of each of these three parameters using the available data.

The following parameters of the 1D model were not directly calculated or estimated from the *in vivo* data: the blood density 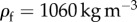 and viscosity 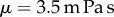 [18], the capillary pressure 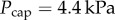 [19] and the polynomial order of the 1D model velocity profile (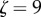) [20]. For the 3D model, in addition to these four parameters, one must further specify values of wall thickness *h*, which was assumed to be 10% of the luminal radius, density of the wall 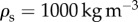, transverse shear factor of the arterial wall 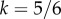 [11] and coefficients related to the stiffness, 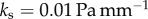 and damping, 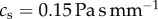, of the external tissue support [11,17].

#### 2.3.1. Elastic mechanical properties

The elastic mechanical properties of the arterial wall were calculated from the pulse wave velocity, *c*. This was estimated from the data using the following two methods.

##### 2.3.1.1. Foot-to-foot method

**Figure 5.**
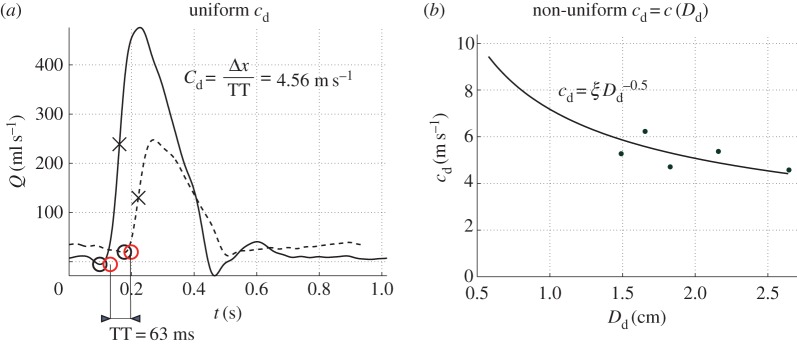
Two different calculations of pulse wave velocity at diastolic pressure, 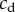, used in this study: (*a*) uniform 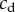 in all 1D model arterial segments calculated using the foot-to-foot method applied to the flow waveforms at the Asc Ao (solid line) and Desc Ao 4 (dashed line); or (*b*) diameter-dependent 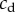 calculated from estimates of 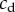 at five aortic sites using the *QA*–loop method [22]. In (*a*), the transit time (TT) was calculated from the time of the foot of each wave, which was identified as the intersection (red circles) of a horizontal projection through the local minimum (black circles) and a tangential projection through the maximum gradient in systole (crosses), as detailed in [21]. The two sites are separated by 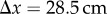 measured along the aortic 3D centreline. In (*b*), 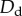 is the luminal diameter at diastolic pressure. The curve is a least-squares fitting of the form 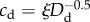 (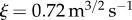) as described in [23].

A uniform 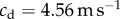 was calculated and mapped to all 1D model segments. This value was obtained as the ratio of the centreline distance between the PC-MRI measurement sites at the ascending aorta and the most distal descending aorta (figure 1*d*) to the transit time of the foot of the flow waveforms at these two sites (figure 2*b*). The foot of the wave was defined using Gaddum *et al.*'s algorithm [21], as shown in figure 5*a*.

##### 2.3.1.2. *QA*–loop method

We applied the algorithm described in [22] to calculate a local 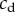 at the five aortic sites where blood flow and luminal area waveforms were measured. Following the approach described in [23], we related 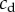 to the local luminal diameter at diastolic pressure, 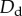, calculated from the *in vivo* area waveforms by assuming a circular cross section (figure 5*b*). We then obtained a least-squares fitting of the form
2.4
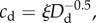
with 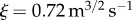. Table 1 shows the values of 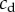 for each of the 1D model arterial segments. Equation (2.4) is in agreement with the form of equation (2.2) for 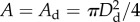; that is 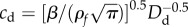. It enables calculation of 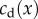 at any point in the 1D domain from 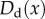 under the assumption of a uniform *β*. Thus, with this approach, we obtain a spatially varying distribution of pulse wave velocities from multiple PC-MRI and area measurements down the aorta.

Once 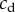 is known, the elastic parameter 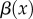 required for the 1D models follows from equation (2.3). For the 3D models, the elastic modulus, 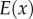, is required instead and was calculated using
2.5
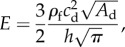
which is obtained by substituting *β* given by equation (2.1) into equation (2.3).

#### 2.3.2. Parameters of the Windkessel outlet models

We calculated the parameters of the four 0D Windkessel models at the outlets of the 1D domain using a linear analysis of the 1D/0D formulation previously presented in [24,25]. This analysis provides analytical equations relating the aortic root inflow wave, 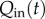, and a space-independent Windkessel pressure, 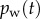, which approximates pressure waves throughout the computational domain. The parameters in these analytical equations are 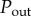 and the wall compliance, 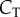, and blood flow resistance, 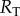, of the entire systemic circulation, which in turn depend on the distributed compliances of the 1D model segments, and peripheral resistances and compliances of the 0D Windkessel models [24]. Appendix B provides further details of all the equations used in this section.

##### 2.3.2.1. Total peripheral resistances

First, the resistance at the root of the 1D/0D arterial network, 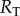, was calculated as
2.6
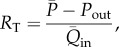
with 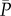 the mean blood pressure of the ensemble averaged carotid waveform (figure 3*a*) and 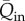 the mean flow rate at the aortic root (figure 2*b*). The outflow pressure 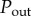 was either set to reported values of capillary pressure, 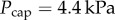 [19], or calculated by fitting a curve of the form
2.7
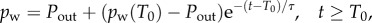
to the ensemble averaged carotid, brachial and radial pressures in diastole (figure 3, red lines), as described in [24]. Here, 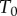 and 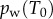 are the time and pressure, respectively, at the beginning of the fit, when pressure starts decaying exponentially, and 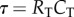 is the time constant of the exponential decay of pressure in diastole. The average values obtained from the three pressures in figure 3 are 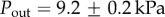 and 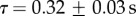.

Then, the total resistance at the outlet of each terminal vessel 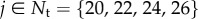, 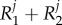, was calculated using
2.8
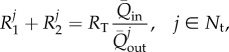
with 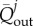 the mean outflow at the outlet of each terminal branch. The ratio of mean flows in equation (2.8) was calculated from the percentages of 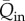 leaving each terminal vessel (figure 2*a*).

##### 2.3.2.2. Peripheral compliances

First, the total peripheral compliance, 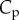, was calculated using
2.9
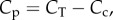
with 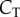 the compliance of the entire 1D/0D model network and 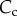 the sum of the compliances of each 1D model segment, 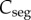, which were calculated using equation (B 6) in appendix B.

For the models in which the diastolic pressure decay was fitted to match the experimental pressure data (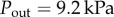), 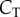 was calculated from the time constant, 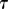, and net resistance, 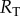, as [24]
2.10
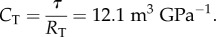


For the models in which the capillary pressure was assumed (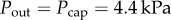), we adopted [24]
2.11
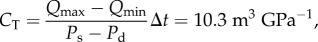
where 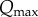 and 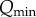 are the maximum and minimum flow rates calculated from the aortic root inflow 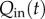 (figure 2*b*), 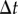 is the difference between the time at 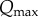 and the time at 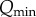, and 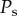 and 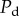 are the systolic and diastolic pressures, respectively, of the ensemble average carotid waveform in figure 3*a*.

Once 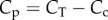 was known, we then calculated 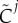, 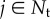, by distributing 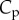 to each terminal branch in proportion to the flow distribution used in equation (2.8) [26], that is
2.12
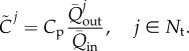
The value of 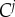 in the Windkessel model, which is different from the resistance-weighted compliance 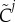, is given by
2.13
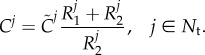


For the models in which 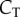 was approximated by equation (2.11), we used the iterative procedure described in [27] to refine the values of 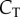 and 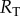 used to calculate 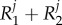 and 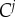, 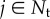.

##### 2.3.2.3. Proximal resistances

The proximal resistance, 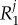, in the three supra-aortic vessels (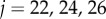) was assumed to be equal to the characteristic impedance, 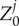, of the end point of the terminal vessel *j*, that is
2.14
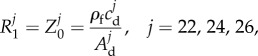
where 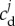 and 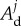 are, respectively, the wave speed and area at diastolic pressure at the end point. This choice of 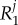 minimizes the magnitude of waves reflected at the outlet of the 1D model upper branches [25].

At the descending aorta outlet (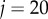), a proximal resistance, 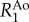, was calculated to reproduce characteristic inflection points of the carotid pressure waveform (figure 3*a*). This modelling assumption implies that the main site of wave reflections in our model was located in the descending aorta, in agreement with clinical observations [28], whereas the upper branches were treated as non-reflective boundaries. The following equation was derived to calculate the value of 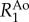 (see appendix C):
2.15
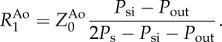

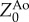
 is the characteristic impedance at the outlet of the descending aorta, computed using equation (2.14) with the parameters of the descending aorta (Vessel 20), 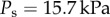 is the systolic pressure of the ensemble average carotid waveform, 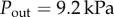, and 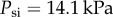 is the pressure at the inflection point during the pressure decay in the second half of systole (figure 3*a*). The inflection point was calculated as the local maximum of the first derivative of the ensemble average carotid waveform. For our volunteer, we obtained 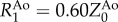 and a negative reflection coefficient of 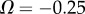 calculated using equation (B 7).

### 2.4. Reconciling data inconsistencies

We made every effort to acquire the *in vivo* data in the volunteer under similar physiological conditions, since we aimed to simulate waveforms for a single physiological state. However, we observed several data inconsistencies in (i) HR, (ii) MRI-derived vessel geometry, and (iii) mean volume flow rates. This section describes how these inconsistencies were addressed to mitigate their impact on the simulation workflow.

#### 2.4.1. Heart rate

The average HR calculated from the flow (61.5 ± 3.4 bpm) and area (65.2 ± 1.5 bpm) measurements was greater than that calculated from the pressure (59.3 ± 2.4 bpm) measurements, possibly because MRI acquisition required the volunteer to be constrained inside the scanner for almost 2 h and to perform several breath holds. The impact of this inconsistency was mitigated by calculating an average HR representative of all *in vivo* measurements (61.2 ± 3.4 bpm). This average HR was then assigned to the aortic inflow waveform (figure 2*b*, colour line).

#### 2.4.2. Magnetic resonance imaging-derived vessel geometry

Differences in diastolic areas were observed between 3D SSFP (used to define the 3D aortic geometry) and 2D SSFP MRI acquisitions (used to generate the dynamic area images). These can be explained by the different spatial resolutions of the 2D and 3D data used. To reduce scanning time, the 3D SSFP anatomy images were acquired with a lower spatial resolution than the 2D cine SSFP dynamic area images (1.5 versus 1.0 mm^3^, respectively). Therefore, we assumed that the 2D data capture better the true diastolic areas and corrected the 3D geometry by matching the diastolic cross-sectional areas given by the five aortic area waveforms in figure 2*c*. This was achieved by (i) computing the ratio of the measured diastolic areas using 2D cine SSFP at the five aortic locations (green contours in figure 1*d*) to the actual cross-sectional areas obtained from slicing the 3D mesh with the measurement planes, (ii) rescaling the 2D contours associated with the aortic 3D mesh, and (iii) regenerating the 3D geometry to match the measured areas in those five locations. The aortic 2D contours in-between measurement locations were scaled with a linearly interpolated scaling factor from the nearest two measurement locations. The 2D contours associated with the arch branches were re-scaled using an average of the scaling factors in the aorta.

#### 2.4.3. Mean volume flow rates

Measures of flow rate by PC-MRI are less exposed to errors in contour segmentation than area measurements. This is because the flow is approximately zero near the arterial wall, and therefore errors in contour segmentation are likely to have a small impact on the overall flow rate. As a result, blood flow data acquired by MRI should be more accurate than luminal area data. However, mean volume flow rates computed from the aortic PC-MRI increased towards the periphery, from Desc Ao 1 to Desc Ao 2 or 3 (figure 2*b*), probably due to the smaller HR recorded in Desc Ao 1 (figure 2*b*). This violates the principle that mean aortic flow decreases as we move down the aorta due to perfusion to small side branches. To mitigate this inconsistency, we calculated the percentage of cardiac output leaving the aorta in equation (2.8) using the mean value among the four aortic flow waves measured by PC-MRI at the locations labelled Desc 1 to Desc 4 in figure 2*a*.

## 3. Results and discussion

We investigated multiple modelling assumptions in which vessel stiffness and boundary condition parameters were defined via the different methods described in §2.3. Using the 1D/0D framework for its computational efficiency, multiple permutations of uniform and non-uniform pulse wave velocity, reflective and matched proximal outflow resistances, and capillary and data-fitted 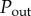 were tested against the available *in vivo* data. These are listed in appendix C. We obtained two different sets of modelling assumptions that produced the smallest relative errors^1^ for aortic area and carotid pressure. Hereafter, we refer to these sets as the ‘best area’ (§3.1) and ‘best pressure’ (§3.2) models. We then studied the impact of uniform versus non-uniform pulse wave velocities (§3.3), and matched versus reflective proximal outflow resistances (§3.4) on these models. Lastly, 3D and 1D simulations were compared for the ‘best area’ and ‘best pressure’ models by using compatible material laws and identical inflow and outflow boundary conditions in both modelling frameworks (§3.5).

### 3.1. Best aortic area waveform predictions

**Figure 6.**
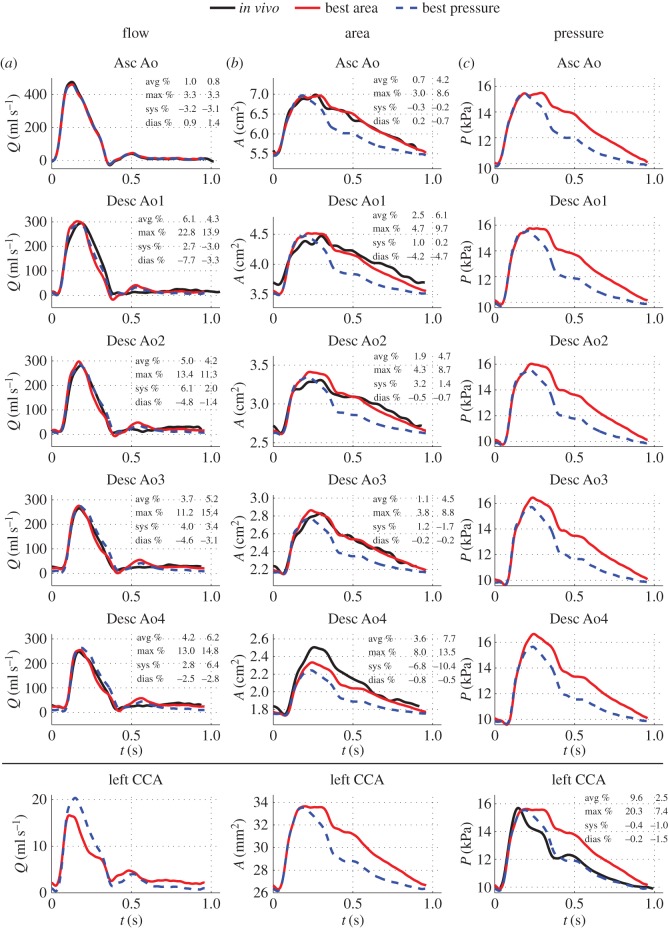
(*a*–*c*) Waveform predictions by the ‘best area’ (colour solid lines) and ‘best pressure’ (colour dashed lines) models in the five aortic sites where MRI measurements were taken (first five rows) and at the outflow of the left common carotid artery (CCA, last row). *In vivo* waveforms (black lines) are shown for the aortic flows, aortic areas and carotid pressure, together with average (avg), maximum (max), systolic (sys) and diastolic (dias) relative error metrics (first column: ‘best area’ model; second column: ‘best pressure’ model).

Figure 6 compares *in vivo* measurements of aortic blood flow, aortic luminal area and carotid blood pressure with corresponding numerical predictions calculated by both the ‘best area’ and ‘best pressure’ 1D models. Aortic pressures and carotid flows and areas are also displayed for both models, though corresponding *in vivo* data were not acquired. In this section, we focus on the results obtained by the ‘best area’ model (colour solid lines), for which all four terminal branches have an outflow pressure (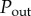) equal to the capillary pressure given in [19] (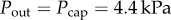), and hence 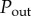 is not specific to our volunteer. The three supra-aortic vessels are coupled to matched Windkessel models (i.e. 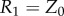), while the descending aorta has a reflective 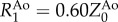 calculated using equation (2.15). Elastic mechanical properties were estimated assuming a uniform pulse wave velocity (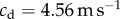) calculated using the foot-to-foot method.

The ‘best area’ model is able to capture well the overall shape of *in vivo* aortic flow and area waves (figure 6*a,b*). The following features are well described by the model: the time and magnitude of the feet of the waves, the amplitude of the flow and area waves, the skewed flow peak in systole, the relatively smaller flow in diastole and the decay in diastolic area. Predictions for aortic flow waveforms show oscillations in systole and early diastole not seen in the *in vivo* measurements. Despite these oscillations, average relative flow errors for the flow do not exceed 7%. Discrepancies in area predictions occur mainly in systole, leading to average relative area errors smaller than 4%.

Systolic and diastolic relative errors for left carotid pressures became smaller than 1% after one iteration of the Windkessel compliances and resistances (figure 6*c*). The simulated carotid pressure wave, however, overestimated the *in vivo* pressure from the systolic peak to the end of the cardiac cycle, leading to an average relative pressure error of almost 10%.

### 3.2. Best carotid pressure waveform prediction

The outflow pressure 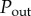 is the only different independent parameter between the ‘best area’ and ‘best pressure’ models: 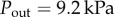 for the ‘best pressure’ model versus 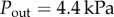 for the ‘best area’ model. Therefore, 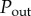 plays a very important role in shaping arterial pulse waveforms (figure 6). Unlike in the ‘best area’ model, 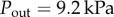 is specific to our volunteer: it is based on an exponential fit of the form given by equation (2.7) to the decay in pressure during diastole in the carotid, brachial and radial arteries (figure 3). We have obtained very similar values of *τ* and 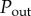 in these three vessels (figure 3) which suggests that late-diastole pressure is approximately uniform in space, in agreement with the derivation of equation (2.7) [24].

The predicted carotid pressure wave by the ‘best pressure’ model features the systolic and diastolic inflection points observed *in vivo*, captures the time and shape of the systolic peak better than the ‘best area’ model and reproduces well the decay in pressure in diastole (figure 6*c*). As a result, average and maximum relative errors for carotid pressure are considerably reduced with respect to the ‘best area’ model. However, aortic areas are not predicted as well as by the ‘best area’ model (figure 6*b*): area is underestimated in all five aortic sites during most of the cardiac cycle by around 10%. Given that average relative errors in *in vivo* aortic flow are smaller for the ‘best pressure’ model from Asc Ao to Desc Ao 2 and for the ‘best area’ model in Desc Ao 3 and 4 (figure 6*a*), we cannot determine the best choice for 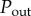 from the *in vivo* data that are available for this study. Invasive *in vivo* aortic pressure waveforms would be required for this purpose.

By correcting the 3D arterial geometry based on the five 2D SSFP area measurements (§2.4.2), we obtained, in both the ‘best area’ and ‘best pressure’ models, relative errors for diastolic areas smaller than 1% in all aortic sites except for Desc Ao 1, where errors were up to 5% (figure 6*b*). This is because the PC-MRI plane selected to acquire the *in vivo* luminal contours at Desc Ao 1 was more oblique than the planes considered at the other four aortic locations (figure 1*d*): indeed, the angle between the MRI contours (shown in green) and the contours on planes perpendicular to the aortic axis used to generate 1D model areas (shown in black) was 17° at Desc Ao 1 and smaller than 9° at the other four aortic sites. Automatic calculation of planes perpendicular to the aortic axis by the MRI acquisition software may, therefore, improve the quality of the PC-MRI data required for subject-specific modelling.

### 3.3. Uniform versus non-uniform pulse wave velocity

**Figure 7.**
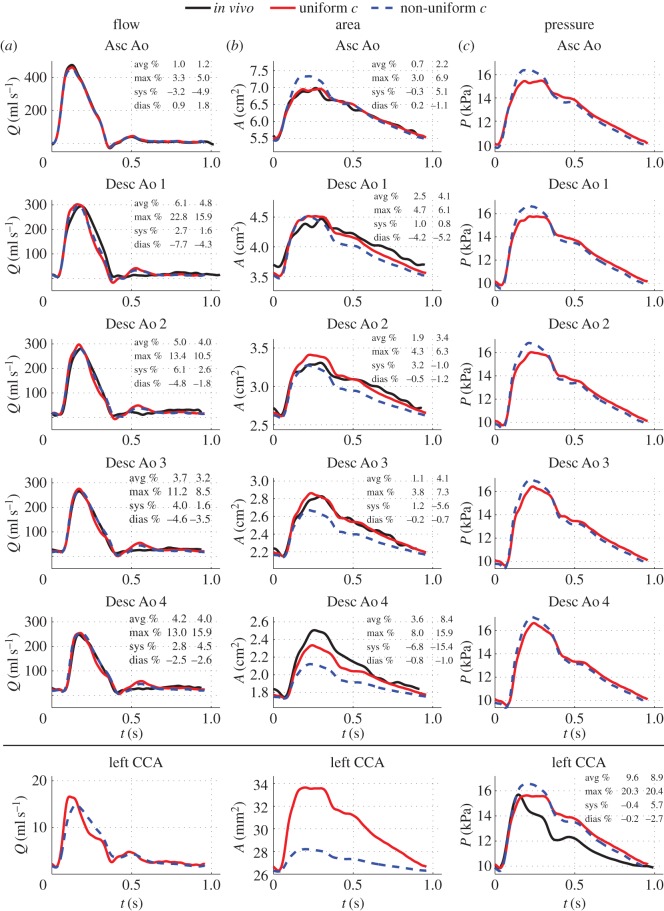
Effects of uniform (colour solid lines) versus non-uniform (colour dashed lines) pulse wave velocity, *c*, on waveforms produced by the ‘best area’ model. Relative errors are for the uniform (first column) and non-uniform (second column) *c* models.

A uniform pulse wave velocity, 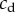, calculated using the foot-to-foot method (figure 5*a*) was used in both ‘best area’ and ‘best pressure’ models to determine the elastic properties of all 1D model arterial segments. Using a non-uniform 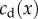 calculated by the *QA*–loop method (figure 5*b*) also predicted well the arrival time of the feet of *in vivo* aortic flow, aortic area and carotid pressure waveforms (figure 7 shows the results for the ‘best area’ model only). However, in both the ‘best area’ and ‘best pressure’ modelling assumptions, a distributed 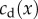 increased relative errors for aortic areas (figure 7*b*). These were consistently underestimated by a non-uniform 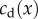, which suggests that this modelling assumption led to an overestimation of aortic stiffness. Indeed, in all arterial segments (except for those in the ascending aorta), non-uniform values of 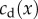 are greater than the uniform 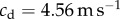 (see table 1), which leads to greater elastic moduli 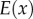 (stiffer walls) according to equation (2.5). Stiffer arterial walls reduced the total compliance compared with the uniform 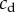 case, preventing the iterative process described in [27] from producing the target *in vivo* pulse pressure and, hence, increasing systolic and diastolic relative errors for the carotid pressure (figure 7*c*).

In the ‘best pressure’ model, we also observed overall larger relative errors for aortic flow when a non-uniform 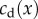 was used. In the ‘best area’ model, relative errors in aortic flow waveforms decreased in descending aorta sites if a non-uniform 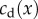 was used (figure 7*a*). In either model, however, the main changes introduced by a non-uniform 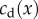 were observed in area and pressure waves of the aorta and carotid artery, rather than in the flow waves. According to these results, the foot-to-foot method provides a better estimate of pulse wave velocity in the upper thoracic aorta than the *QA*–loop method. Indeed, considerable relative errors (over 30%) in the estimates of *c* obtained by the *QA*–loop method have been reported in [29,30].

### 3.4. Matched versus reflective proximal outflow resistances

**Figure 8.**
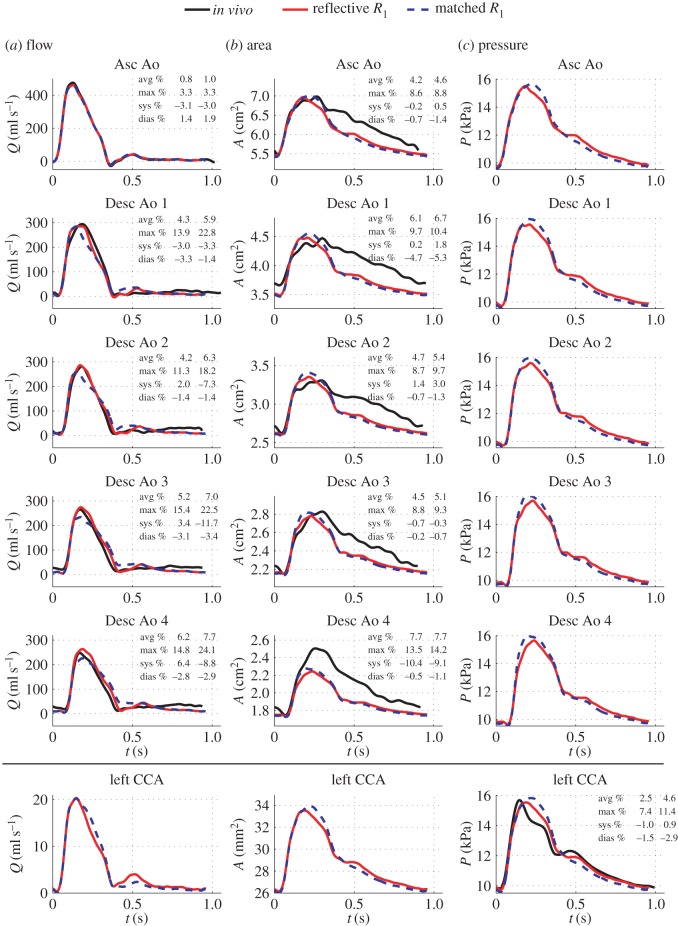
Effects of reflective (colour solid lines) versus well-matched (colour dashed lines) aortic resistance 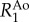 on waveforms produced by the ‘best pressure’ model. Relative errors are for the reflective (first column) and well-matched (second column) 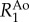 models.

An important result of our study is the fact that a reflective resistance 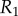 was required at the outflow of the descending aorta (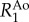) to improve the accuracy of flow, area and pressure waveforms produced by the 1D/0D formulation. Figure 8 compares aortic and carotid flows, areas and pressures calculated by the ‘best pressure’ model, with a reflective aortic 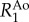 computed using equation (2.15), with the corresponding waveforms simulated using a well-matched 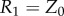 in all four terminal branches. A reflective 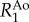 had the opposite effect on aortic flows to aortic pressures/areas (figure 8): it increased systolic flows and decreased systolic pressures/areas, leading to smaller average relative errors than those produced by a matched 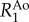. The effect of the reflective 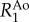 is in agreement with the linear 1D pulse wave theory used to obtain equation (2.15): the theory described in [25] shows that a negative reflection coefficient at a 1D model terminal end (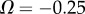 in our case) will decrease the amplitude of reflected pressure wavefronts and will increase the amplitude of reflected flow wavefronts. Moreover, a reflective 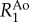 was required to generate the diastolic pressure peak observed *in vivo* (figure 8*c*), which suggests that reflected waves in the lower body travelling upstream of the aorta play an important role in shaping aortic and carotid pulse waveforms. This result is consistent with clinical observations [28].

Similar results were obtained when comparing matched versus reflective 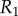 for the ‘best area’ model (not shown). Lastly, we note that using equation (2.15) to calculate 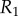 in the three supra-aortic vessels had an insignificant effect on the waveforms produced by the ‘best area’ and ‘best pressure’ models.

### 3.5. One-dimensional versus three-dimensional haemodynamics

**Figure 9.**
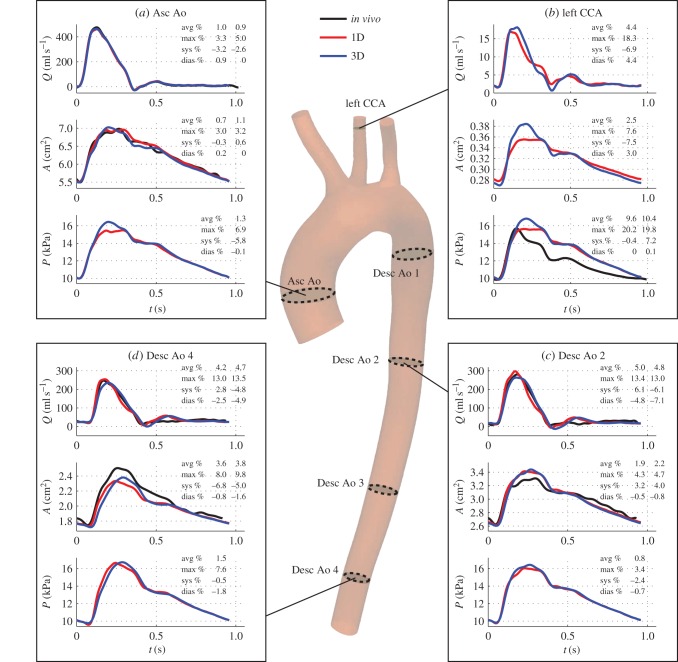
Blood flow (*Q*), luminal area (*A*) and blood pressure (*P*) waveforms computed using the 1D (red lines) and 3D (blue lines) ‘best area’ models in four arterial sites: (*a*) ascending aorta, (*b*) left common carotid artery (CCA) (one diameter away from the outlet), (*c*) descending aorta 2 and (*d*) descending aorta 4. Available *in vivo* waveforms at these sites are shown in black lines. Errors are shown for the 1D model (first column) and the 3D model (second column) relative to the *in vivo* data, if available; else only for the 1D model relative to the 3D model.

**Figure 10.**
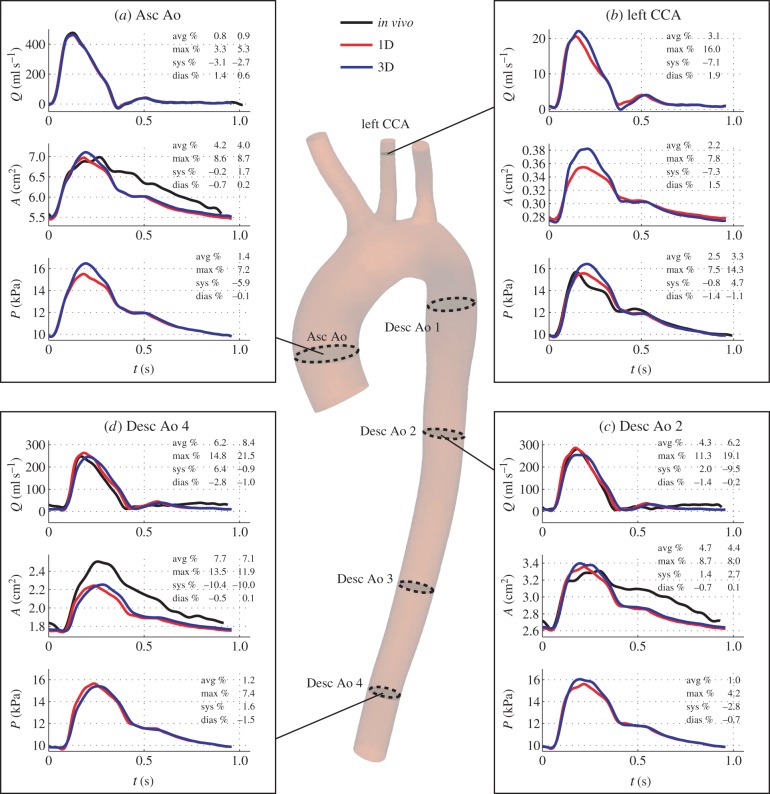
Results for the ‘best pressure’ modelling assumptions (same format as in figure 9).

We performed a comparison between 1D and 3D results for the ‘best area’ and ‘best pressure’ modelling assumptions. In general, 1D and 3D theories produced similar flow, area and pressure waveforms with comparable relative errors at sites where *in vivo* data were available. Figure 9 displays 1D and 3D results computed under the ‘best area’ assumptions, together with *in vivo* waveforms, at the Asc Ao, left CCA, Desc Ao 2 and Desc Ao 4 locations. Figure 10 shows the corresponding results under the ‘best pressure’ assumptions. Simulated aortic flow, aortic area and carotid pressure waveforms using 1D and 3D modelling were both able to capture the main features of corresponding *in vivo* waveforms. This is confirmed by average relative errors smaller than 7%, 4% and 11%, respectively, using the ‘best area’ assumptions and smaller than 9%, 8% and 4%, respectively, using the ‘best pressure’ assumptions. Such small errors suggest that subject-specific 1D/0D and 3D/0D models are both able to capture the main features of *in vivo* aortic flow, aortic area and carotid pressure waveforms under normal anatomical and physiological conditions.

*In vivo* measurements were not available to test the accuracy of simulated aortic pressures, carotid flow and carotid area waveforms. For these waveforms, we compared 1D model against 3D model predictions. Previous studies [5,27] had carried out 1D versus 3D comparisons in idealized geometries, but not in subject-specific geometries as we have done in this study. Our results showed that simulated aortic pressures, carotid flow and carotid area waves using the 1D formulation contained the main features of corresponding 3D model waves. Average relative errors were smaller than 2%, 5% and 3%, respectively, in both the ‘best area’ and ‘best pressure’ models, which are similar to corresponding errors obtained in idealized geometries [5,27]. The greatest discrepancies were observed in mid-systole, as we had previously observed in idealized geometries [27]: the 1D formulation underestimates 3D model systolic pressures along the aorta, with differences becoming smaller towards the periphery. A greater pressure gradient is therefore necessary between the root and outflow of the aorta in the 3D model during mid-systole. This indicates that more energy is dissipated along the 3D aortic model: part of the energy is consumed in driving secondary flow patterns that develop in the aortic arch due to the curvature of the vessel and which are not captured by the 1D formulation. The small relative errors between 1D and 3D modelling approaches suggest that our combined 1D/0D method for calculating mechanical properties and outflow boundary conditions for 3D aortic flow modelling—introduced in [27] for idealized aortic and carotid geometries—can also be applied to subject-specific geometries under normal physiological and anatomical conditions; for example in the absence of occlusive or aneurysmal disease.

We note that the overall shape of the flow waveform in the left common carotid artery obtained by the ‘best area’ and ‘best pressure’ models is qualitatively similar to that measured with PC-MRI in [6] for a younger normal subject. The predicted flow is unidirectional for the entire cardiac cycle and contains a characteristic peak in early diastole (figures 9*b* and 10*b*).

Previous studies had verified 1D and 3D model waveforms by comparison against *in vivo* data in more extensive vascular networks than the thoracic aorta used here [3,6,31]. While including additional arteries will make the computational domain more complete, it also makes it more challenging to personalize all modelling parameters to a given subject. In previous studies, numerous parameters had to be obtained from the literature or tuned; for example using optimization techniques [17]. As a result, only a small proportion of the total number of parameters could be determined from the available *in vivo* data. Here we have minimized the number of arbitrary parameters by focusing on a confined region of the systemic vasculature and acquiring a rich array of non-invasive MRI and tonometry data. Therefore, all parameters of the aortic 1D/0D models were directly calculated or estimated from the data, except for the blood density 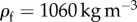 and viscosity 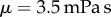 [18], the polynomial order 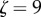 of the velocity profile [20], and, for the ‘best area’ model, the capillary pressure 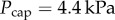 [19]. For the 3D/0D models, in addition to these four parameters, we had to arbitrarily specify values of wall density and thickness, mechanical properties of external tissue support, and transverse shear factor.

Lastly, we note that blood viscosity had a small effect on the aortic waveforms computed by the 1D model: the inviscid 1D solution changed average relative errors for flow, area and pressure waveforms by less than 0.4% in both the ‘best area’ and ‘best pressure’ models (see appendix C). According to this result, the inviscid 1D solution could also be used to simulate pulse wave propagation in the upper thoracic aorta, under normal anatomical and physiological conditions. In this scenario, we avoided estimating blood viscosity and the shape of the velocity profile, reducing to two the number of 1D model parameters that are not subject specific for the ‘best area’ model (the blood density, 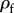, and capillary pressure, 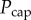) and to only one parameter (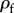) for the ‘best pressure’ model.

## 4. Clinical implications

The 1D/0D methodology described in this study enables calculation of pressure, flow and area waves in the upper aorta and supra-aortic vessels from non-invasive measurements in a discrete number of locations. It provides a means of calculating patient-specific estimates of haemodynamic quantities that are relevant to assess cardiovascular function, such as aortic (central) blood pressure [32], aortic pulse wave velocity [33] and wall shear stress [18]; the latter using the 3D/0D formulation with parameters determined by the 1D/0D methodology. However, the 1D/0D algorithm has only been verified in one subject and using non-invasive data. Further verification in a clinical cohort is, therefore, essential before our method could become a clinical tool for non-invasive detailed characterization of aortic haemodynamics. Verification against intra-vascular aortic pressures will allow us to test aortic pressure predictions and elucidate whether the ‘best area’ model provides more accurate results than the ‘best pressure’ model or *vice versa*. Clinical cohorts with different cardiovascular pathologies should also be investigated, for example, to determine the applicability limits of this 1D/0D method in anatomies such as aortic coarctation or aneurysms.

In §2.4, we have discussed the importance of reconciling several inconsistencies in the clinical data used for calibrating our 1D and 3D models. In addition, the impact of uncertainties affecting the measured data should be analysed before translating the method to the clinic. For example, uncertainties in the flow split at the supra-aortic vessels measured by PC-MRI have been shown to have a significant effect on wall shear stress-based indicators [34] and may also affect considerably the pattern of predicted pressure, flow and area waveforms.

## 5. Conclusion

We have shown that accurate, subject-specific, 1D/0D and 3D/0D models of pulse wave haemodynamics in the upper aorta of a young healthy volunteer can be obtained using non-invasive clinical data. By simulating blood flow in a confined region of the systemic vasculature and acquiring a substantial amount of *in vivo* measurements, we have minimized the number of arbitrary modelling assumptions and determined most of the model parameters from the *in vivo* data. We have provided a detailed workflow for calculating the geometrical and mechanical properties of the computational domains, as well as outflow boundary conditions, from non-invasive data acquired by MRI and tonometry. The following are key aspects of this workflow to minimize relative errors for aortic flows, aortic areas and carotid pressures computed using the 1D/0D formulation: (i) elimination of inconsistencies in the clinical data, (ii) a uniform pulse wave velocity calculated from PC-MRI flow waves by the foot-to-foot method, (iii) a reflective resistance at the outflow of the descending aorta calculated from the shape of the carotid pressure wave, and (iv) an outflow pressure either equal to the capillary pressure to better predict aortic area waves or calculated from the tonometry pressure waves to better predict the carotid pressure wave. We have also demonstrated good agreement between 1D and 3D model predictions, especially during early systole and diastole. This study supports the use of 1D and 3D models for subject-specific modelling of aortic pulse wave haemodynamics, as well as the use of the 1D/0D formulation for an efficient calculation of parameters for 3D/0D modelling.

## Supplementary Material

Aortic 3-D volume mesh

## Supplementary Material

In Vivo aortic flow waveforms

## Supplementary Material

In Vivo aortic area waveforms

## Supplementary Material

In Vivo pressure waveforms

## Endnote

1 Relative errors for simulated area, flow and pressure were calculated as described in [27].

## Data accessibility

The *in vivo* datasets supporting this article have been uploaded as part of the electronic supplementary material.

## Appendix A. Data acquisition technology

Table 2 provides a brief description of the non-invasive techniques used in this study to acquire the *in vivo* data, focusing on the type of haemodynamic data measured by each technique.

**Table 2.** Brief description of the data acquisition technology.

concept	description
1. SSFP MRI	steady-state free precession is a type of MRI pulse sequence noted for its superiority in dynamic/cine images. It was used to acquire the aortic 3D geometry (figure 2*a*) and area waveforms (figure 2*c*)
2. PC-MRI	phase-contrast MRI relates proton movement to blood velocity. Aortic blood flows (figure 2*b*) were obtained by multiplying blood velocities by luminal cross-sectional areas segmented using GTFlow (GyroTools LLC)
3. cine images	series of frames covering one full period of cardiac cycle that can be displayed as a movie (cine). Each frame of the 2D cine SSFP dynamic area images used to produce the aortic area waveforms (figure 2*c*) was composed of information gathered over several heart beats
4. flash angiography	MRI sequence for rapid visualization of the lumen of blood vessels without a substantial loss in image quality
5. gated MRI	the MRI signal acquisition is synchronized to the cardiac or respiratory cycle. The R-wave of the electrocardiogram (ECG) was used as a reference to align in time the PC-MRI flow and 2D cine SSFP area waveforms shown in figure 2
6. non-contrast MRI	MRI images were not enhanced by invasive injection of contrast material
7. TE	time to echo, measured in milliseconds, is the time between the application of the radiofrequency excitation pulse and the peak of the signal induced in the coil
8. TR	time to repetition, measured in milliseconds, is the time from the application of an excitation pulse to the application of the next pulse
9. applanation tonometry	blood pressure was measured from the force required to flatten (applanate) a constant area of the carotid, brachial or radial arteries

## Appendix B. Windkessel model parameter estimation

A linear analysis of the governing 1D/0D equations used in this study provides analytical equations describing the effect of model parameters on pressure, flow and area waveforms [24,25]. This appendix describes those analytical equations that were used in §2.3.2 to calculate the parameters , *C*,  and  of each Windkessel outlet model (figure 4) from the *in vivo* pressure and flow waves.

**B.1. Total peripheral resistances**

By assuming periodic flow and neglecting nonlinearities and viscous dissipation in the 1D model segments of an arterial network coupled to three-element Windkessel outlet models (figure 4), the time-average pressure over one cardiac cycle, , measured in any 1D model segment (the aorta and supra-aortic vessels in this study) is given by [24]

B 1

 is the pressure at the outflow of each Windkessel model, assumed to be the same at each outlet,  is the resistance at the root of the 1D/0D arterial network, which depends on the individual resistances of the 0D Windkessel models,  is the set of arterial segments that are terminal branches and  is the time-averaged inflow waveform. For the upper thoracic aorta considered in this study,  is the mean flow rate at the ascending aorta (figure 2*b*) and  (figure 1*e*). Rearranging equation (B 1) and taking *P* to be the pressure wave at the carotid artery leads to equation (2.6). By applying equation (B 1) to a network consisting of a single 1D model vessel (each terminal branch  in this study), we can write

B 2

with  the mean flow at the outlet of the vessel (which is equal to the mean flow at the inlet for a single vessel). Equation (2.8) follows from combining equations (B 1) and (B 2) and solving for .

As also detailed in [24], pressure waves in any 1D model segment of the network can be approximated by a space-independent 0D Windkessel pressure, , which depends on the 1D/0D model parameters of the network. This is achieved by neglecting nonlinearities, blood flow inertia and viscous dissipation in 1D model segments. Under these assumptions,  is given by

B 3

where  is the pressure  at a reference time ,  is the compliance of the entire 1D/0D arterial network,  is the inflow waveform and  is the outflow at each terminal branch. For the upper thoracic aorta considered in this study,  is the blood flow at the ascending aorta shown in figure 2*b* (colour line). Figure 3*a* shows the space-independent pressure, , calculated using the parameters of the ‘best pressure’ model, with  corresponding to the time at the start of systole.

By taking  to be the time when pressure starts decaying exponentially in diastole, and assuming  for , —note that  is approximately zero for  (figure 2*b*)—equation (B 3) reduces to equation (2.7), with .

**B.2. Peripheral compliances**

The total compliance  consists of both 1D and 0D compliances [24]:

B 4

where  is the total compliance of the 1D model arterial network and  is the total peripheral compliance. Equation (B4) can be rearranged in the form of equation (2.9). The compliances  and  depend on, respectively, the compliance of each 1D model segment () and the parameters of the 0D Windkessel outlet models (,  and , ) through

B 5

with *N* the total number of segments in the 1D model arterial network ( in this study) and  the resistance-weighted compliance of each Windkessel outlet model. Rearranging the third expression in equation (B5) yields equation (2.13). The compliance  of each 1D model segment can be calculated as

B 6

with *L* the segment length, and  and , respectively, the area and wave speed at diastolic pressure.

**B.3. Proximal resistances**

The linear analysis presented in [25] shows that a pressure wavefront, , propagating towards the outlet of a straight 1D model arterial segment coupled to a single-resistance outlet, , produces a reflected pressure wavefront, , given by

B 7

Ω is the terminal reflection coefficient and  is the characteristic impedance of the 1D model segment. According to equation (B 7), the incoming pressure wavefront is completely absorbed if , leading to equation (2.14) for calculating  in the three supra-aortic vessels.

At the outlet of the descending aorta, a reflective  was calculated by assuming that the amplitude at the inflection point, , of the carotid pressure waveform (figure 3*a*) is caused by the reflection at the aortic outlet of a wave of amplitude  propagating from the ascending aorta in early systole. Thus, equation (2.15) follows from taking , ,  and  in equation (B 7).

## Appendix C. Parameter permutations

Table 3 lists all the different permutations of pulse wave velocity, *c*, proximal aortic outflow resistance, , and terminal outflow pressure, , that were tested against the available *in vivo* data using the 1D/0D framework. For each set of modelling assumptions, average relative errors between *in vivo* and predicted waveforms are provided at the same arterial sites as in figure 6. The last two rows show the results for the ‘best pressure’ and ‘best area’ models under the assumption of inviscid flow; that is .

**Table 3.** Modelling assumptions and average relative errors for all tested permutations. The minimum average relative errors among all modelling assumptions are highlighted in bold.

permutation	modelling assumptions			average relative errors		
	pulse wave velocity	aortic outflow resistance	terminal outflow pressure	aortic flows Asc–Desc I–II–III–IV	aortic areas Asc–Desc I–II–III–IV	carotid pressure
1. ‘best area’ (figure 6)	uniform	reflective	capillary	1.0–6.1–5.0–3.7–4.2	**0.7–2.5–1.9–1.1–3.6**	9.6
2. ‘best pressure’ (figure 6)	uniform	reflective	data-fitted	**0.8**–4.3–4.2–5.2–6.2	4.2–6.1–4.7–4.5–7.7	**2****.****5**
3. ‘best area’ with non-uniform *c* (figure 7)	non-uniform	reflective	capillary	1.2–4.8–**4.0–3.2–4.0**	2.2–4.1–3.4–4.1–8.4	8.9
4. ‘best pressure’ with non-uniform *c*	non-uniform	reflective	data-fitted	1.1–**3.4**–5.0–6.5–8.2	5.4–7.3–6.3–6.8–10.8	4.8
5. ‘best area’ with well-matched	uniform	well-matched	capillary	1.1–6.2–5.3–4.5–5.0	1.1–3.2–2.5–1.5–**3.6**	9.7
6. ‘best pressure’ with well-matched (figure 8)	uniform	well-matched	data-fitted	1.0–5.9–6.3–7.0–7.7	4.6–6.7–5.4–5.1–7.7	4.6
7. combination of 3. and 5.	non-uniform	well-matched	capillary	1.5–5.9–6.4–5.7–6.0	3.3–4.8–3.7–4.0–8.4	9.8
8. combination of 4. and 6.	non-uniform	well-matched	data-fitted	1.4–6.4–7.7–8.9–9.6	7.0–8.1–6.7–6.9–10.8	8.3
9. inviscid ‘best area’	uniform	reflective	capillary	1.0–6.3–5.3–4.0–4.5	**0.7–2.5–1.9–1.1–3.6**	9.4
10. inviscid ‘best pressure’	uniform	reflective	data-fitted	**0.8**–4.4–4.3–5.4–6.4	4.4–6.2–4.8–4.6–7.7	**2****.****5**

## References

[1] Westerhof N, Lankhaar JW, Westerhof BE 2009 The arterial Windkessel. Med. Biol. Eng. Comput. 47, 131–141. (10.1007/s11517-008-0359-2) 18543011

[2] Ursino M 1998 Interaction between carotid baroregulation and the pulsating heart: a mathematical model. Am. J. Heart Circ. Physiol. 275, H1733–H1747. 10.1152/ajpheart.1998.275.5.H1733 9815081

[3] Olufsen MS, Peskin CS, Kim WY, Pedersen EM, Nadim A, Larsen J 2000 Numerical simulation and experimental validation of blood flow in arteries with structured-tree outflow conditions. Ann. Biomed. Eng. 28, 1281–1299. (10.1114/1.1326031) 11212947

[4] Formaggia L, Lamponi D, Quarteroni A 2003 One-dimensional models for blood flow in arteries. J. Eng. Math. 47, 251–276. (10.1023/B:ENGI.0000007980.01347.29)

[5] Mynard JP, Nithiarasu P 2008 A 1D arterial blood flow model incorporating ventricular pressure, aortic valve and regional coronary flow using the locally conservative Galerkin (LCG) method. Commun. Numer. Meth. Eng. 24, 367–417. (10.1002/cnm.1117)

[6] Reymond P, Bohraus Y, Perren F, Lazeyras F, Stergiopulos N 2011 Validation of a patient-specific one-dimensional model of the systemic arterial tree. Am. J. Physiol. Heart Circ. Physiol. 301, H1173–H1182. (10.1152/ajpheart.00821.2010) 21622820

[7] Alastruey J, Parker KH, Sherwin SJ 2012 Arterial pulse wave haemodynamics. In *11th Int. Conf. Pressure Surges* (ed. S Anderson), pp. 401–442. Bedford, UK: British Hydromechanics Research (BHR) Group.

[8] Perktold K, Rappitsch G 1995 Computer simulation of local blood flow and vessel mechanics in a compliant carotid artery bifurcation model. J. Biomech. 28, 845–856. (10.1016/0021-9290(95)95273-8) 7657682

[9] Taylor CA, Hughes TJR, Zarins CK 1998 Finite element modeling of blood flow in arteries. Comput. Meth. Appl. Mech. Eng. 158, 155–196. (10.1016/S0045-7825(98)80008-X)

[10] Quarteroni A, Tuveri M, Veneziani A 2000 Computational vascular fluid dynamics: problems, models and methods. Comput. Visual Sci. 2, 163–197. (10.1007/s007910050039)

[11] Figueroa CA, Vignon-Clementel I, Jansen KE, Hughes TJR, Taylor CA 2006 A coupled momentum method for modeling blood flow in three-dimensional deformable arteries. Comput. Meth. Appl. Mech. Eng. 195, 5685–5706. (10.1016/j.cma.2005.11.011)

[12] Alastruey J, Khir AW, Matthys KS, Segers P, Sherwin SJ, Verdonck PR, Parker KH, Peiró J 2011 Pulse wave propagation in a model human arterial network: assessment of 1-D visco-elastic simulations against *in vitro* measurements. J. Biomech. 44, 2250–2258. (10.1016/j.jbiomech.2011.05.041) 21724188 PMC3278302

[13] Kung EO, Les AS, Figueroa CA, Medina F, Arcaute K, Wicker RB, McConnell MV, Taylor CA 2011 *In vitro* validation of finite element analysis of blood flow in deformable models. Ann. Biomed. Eng. 39, 1947–1960. (10.1007/s10439-011-0284-7) 21404126 PMC4404701

[14] Bushberg J, Seibert J, Leidholdt E, Boone J 2001 The essential physics of medical imaging, 2nd edn Baltimore, MD: Williams and Wilkins.

[15] Hoskins P, Martin K, Thrush A 2010 Diagnostic ultrasound: physics and equipment, 2nd edn Cambridge, UK: Cambridge University Press.

[16] Cuomo F, Ferruzzi J, Humphrey JD, Figueroa CA 2015 An experimental–computational study of catheter induced alterations in pulse wave velocity in anesthetized mice. Ann. Biomed. Eng. 43, 1555–1570. (10.1007/s10439-015-1272-0) 25698526 PMC4497847

[17] Moireau P, Xiao N, Astorino M, Figueroa CA, Chapelle D, Taylor CA, Gerbeau J-F 2012 External tissue support and fluid-structure simulation in blood flows. Biomech. Model. Mechanobiol. 11, 1–18. (10.1007/s10237-011-0289-z) 21308393

[18] Caro CG, Pedley TJ, Schroter RC, Seed WA 1978 The mechanics of the circulation. Oxford, UK: Oxford University Press.

[19] Parazynski SE, Tucker BJ, Aratow M, Crenshaw A, Hargens AR 1993 Direct measurement of capillary blood pressure in the human lip. J. Appl. Physiol. 74, 946–950. 8458818 10.1152/jappl.1993.74.2.946

[20] Hunter P 1972 Numerical solution of arterial blood flow. Auckland, New Zealand: University of Auckland.

[21] Gaddum NR, Alastruey J, Beerbaum P, Chowienczyk P, Schaeffter T 2013 A technical assessment of pulse wave velocity algorithms applied to non-invasive arterial waveforms. Ann. Biomed. Eng. 41, 2617–2629. (10.1007/s10439-013-0854-y) 23817766

[22] Rabben SI, Stergiopulos N, Hellevik LR, Smiseth OA, Slørdahl S, Urheim S, Angelsen B 2004 An ultrasound-based method for determining pulse wave velocity in superficial arteries. J. Biomech. 37, 1615–1622. (10.1016/j.jbiomech.2003.12.031) 15336937

[23] Alastruey J, Nagel SR, Nier B, Hunt AAE, Weinberg PD, Peiró J 2009 Modelling pulse wave propagation in the rabbit systemic circulation to assess the effects of altered nitric oxide synthesis. J. Biomech. 42, 2116–2123. (10.1016/j.jbiomech.2009.05.028) 19646697

[24] Alastruey J, Passerini T, Formaggia L, Peiró J 2012 Physical determining factors of the arterial pulse waveform: theoretical analysis and estimation using the 1-D formulation. J. Eng. Math. 77, 19–37. (10.1007/s10665-012-9555-z)

[25] Alastruey J, Parker KH, Peiró J, Sherwin SJ 2008 Lumped parameter outflow models for 1-D blood flow simulations: effect on pulse waves and parameter estimation. Commun. Comput. Phys. 4, 317–336.

[26] Stergiopulos N, Young DF, Rogge TR 1992 Computer simulation of arterial flow with applications to arterial and aortic stenoses. J. Biomech. 25, 1477–1488. (10.1016/0021-9290(92)90060-E) 1491023

[27] Xiao N, Alastruey J, Figueroa CA 2014 A systematic comparison between 1-D and 3D hemodynamics in compliant arterial models. Int. J. Numer. Meth. Biomed. Eng. 30, 204–231. (10.1002/cnm.2598) PMC4337249 24115509

[28] Chowienczyk PJ, Kelly R, MacCallum H, Millasseau SC, Andersson T, Gosling RG, Ritter JM, Änggård EE 1999 Photoplethysmographic assessment of pulse wave reflection: blunted response to endothelium-dependent *β*_2_-adrenergic vasodilation in type 2 diabetes. J. Am. Coll. Cardiol. 34, 2007–2014. (10.1016/S0735-1097(99)00441-6) 10588217

[29] Alastruey J 2011 Numerical assessment of time-domain methods for the estimation of local arterial pulse wave speed. J. Biomech. 44, 885–891. (10.1016/j.jbiomech.2010.12.002) 21211799 PMC3111821

[30] Swillens A, Lanoye L, Backer JD, Stergiopulos N, Verdonck PR, Vermassen F, Segers P 2008 Effect of an abdominal aortic aneurysm on wave reflection in the aorta. IEEE Trans. Biomed. Eng. 55, 1602–1611. (10.1109/TBME.2007.913994) 18440906

[31] Les AS, Shadden SC, Figueroa CA, Park JM, Tedesco MM, Herfkens RJ, Dalman RL, Taylor CA 2015 Quantification of hemodynamics in abdominal aortic aneurysms during rest and exercise using magnetic resonance imaging and computational fluid dynamics. Ann. Biomed. Eng. 38, 1288–1313. (10.1007/s10439-010-9949-x) PMC6203348 20143263

[32] Vennin S, Mayer A, Li Y, Fok H, Clapp B, Alastruey J, Chowienczyk P 2015 Non-invasive calculation of the aortic blood pressure waveform from the flow velocity waveform: a proof of concept. Am. J. Physiol. Heart Circ. Physiol. 309, 969–976. (10.1152/ajpheart.00152.2015) PMC4591398 26163442

[33] Vlachopoulos C, Aznaouridis K, Stefanadis C 2010 Prediction of cardiovascular events and all-cause mortality with arterial stiffness: a systematic review and meta-analysis. J. Am. Coll. Cardiol. 55, 1318–1327. (10.1016/j.jacc.2009.10.061) 20338492

[34] Gallo D et al. 2012 On the use of *in vivo* measured flow rates as boundary conditions for image-based hemodynamic models of the human aorta: implications for indicators of abnormal flow. Ann. Biomed. Eng. 40, 729–741. (10.1007/s10439-011-0431-1) 22009313

